# Epidemiological associations with genomic variation in SARS-CoV-2

**DOI:** 10.1038/s41598-021-02548-w

**Published:** 2021-11-26

**Authors:** Ali Rahnavard, Tyson Dawson, Rebecca Clement, Nathaniel Stearrett, Marcos Pérez-Losada, Keith A. Crandall

**Affiliations:** 1 Computational Biology Institute, Department of Biostatistics and Bioinformatics, Milken Institute School of Public Health, The George Washington University, Washington, DC USA; 2 CIBIO-InBIO, Centro de Investigação em Biodiversidade e Recursos Genéticos, Universidade do Porto, Campus Agrário de Vairão, Vairão, Portugal

**Keywords:** Computational models, Phylogeny, Statistical methods, Phylogenetics, Data mining

## Abstract

SARS-CoV-2 (CoV) is the etiological agent of the COVID-19 pandemic and evolves to evade both host immune systems and intervention strategies. We divided the CoV genome into 29 constituent regions and applied novel analytical approaches to identify associations between CoV genomic features and epidemiological metadata. Our results show that nonstructural protein 3 (nsp3) and Spike protein (S) have the highest variation and greatest correlation with the viral whole-genome variation. S protein variation is correlated with nsp3, nsp6, and 3′-to-5′ exonuclease variation. Country of origin and time since the start of the pandemic were the most influential metadata associated with genomic variation, while host sex and age were the least influential. We define a novel statistic—coherence—and show its utility in identifying geographic regions (populations) with unusually high (many new variants) or low (isolated) viral phylogenetic diversity. Interestingly, at both global and regional scales, we identify geographic locations with high coherence neighboring regions of low coherence; this emphasizes the utility of this metric to inform public health measures for disease spread. Our results provide a direction to prioritize genes associated with outcome predictors (e.g., health, therapeutic, and vaccine outcomes) and to improve DNA tests for predicting disease status.

## Introduction

The COVID-19 pandemic, caused by the 2019 novel coronavirus (SARS-CoV-2), has fundamentally changed the world. More than a year later, there are a diversity of efforts ongoing to develop both therapeutic strategies as well as vaccine distribution. Yet, we know from experience, the virus will evolve strategies to escape both host immune systems and intervention strategies. Characterizing the dynamics of coronavirus genomic variation is crucial to understand both viral (e.g., virulence) and host (e.g., immune response) biological activities related to these changes, which makes the coronavirus (CoV) genome the most important and most challenging source to investigate the virus behavior. For example, the viral Spike protein is considered a key element of the virus to initiate binding to host cells via cell-surface protein angiotensin-converting enzyme 2 (ACE2)^1,2^; hence, *ACE2* genetic variation has been targeted as a source to explain disease severity^3–5^. Genomic variation has also been shown to be informative in terms of tracking the spread of the virus^6^ and identifying major clades related to different variants of SARS-CoV-2 with different epidemiological features^7^. CoV genomic variation has also been associated with host phenotypic variables (including epidemiological information) via changes in the virus protein structure, giving the latter great potential explanatory power with respect to clinical outcomes^8–10^. Population-level studies of protein structural changes in CoV genomes may likewise inform its epidemic kinetics (e.g., speed of spread). However, our ability to effectively link genomic variation in viruses to epidemiological information is hindered by analytical limitations in current methodologies to test such associations. Here we apply our novel multi-resolution clustering approach for identifying variable CoV genomic regions and linking these regions to epidemiological factors. We apply a coherence metric to quantify and compare phylogenetic divergence of CoV genomes within locations to the divergence of CoV between locations. Our results have direct public health applicability, and the underlying methodology is robust to any collection of genomic features and epidemiological data.

## Results

We applied novel analytical approaches to characterize the dynamic nature of mutations across the coronavirus genome regions and to test for associations with publicly available clinical variables. We used two sets of data: one to investigate SARS-CoV-2 differences against previously detected coronaviruses at the nucleotide and protein level, and another including only SARS-CoV-2 genomes to characterize the dynamics of the COVID-19 pandemic in association with epidemiological data. We generated dissimilarity matrices between the whole viral genome and specific regions, and applied *omeClust*^11^ (“Methods”), a multi-resolution clustering approach to investigate viral lineage diversity in relation to clinical and epidemiological data. We also used the nucleotide-based distance structure among samples to assess the relationship between variation explained by the whole genome and specific regions. Using this approach, we identified novel associations between the spike protein and nsp3 and the whole genome variation within the SARS-CoV-2 and among lineages of other coronaviruses. Further, we found that epidemiological variables such as country of origin and days from the start of the pandemic explain most of the genomic variation. Our results show that host, infected individuals, gender, and age have the lowest explanatory power of the viral genomic variation. This suggests that the viral genome mutations are independent of those specific characteristics of the infected hosts. In addition, the specific gene differences among the coronavirus families drive most of the genome differences, which can explain the speed of spread and higher infectivity of SARS-CoV-2.

### CoV phylogenomics using specific viral genes

We used phylogenetic-based approaches that compute nucleotide similarities to investigate population-level dynamics of viruses; such tools have been previously used to investigate the origin of coronaviruses that infect humans^7,12^. We used phylodynamic techniques to characterize the evolutionary dynamics of COVID-19. We first investigated the detailed genomic variation among coronaviruses to identify important changes at specific regions (i.e., protein and single nucleotide polymorphisms) in relation to epidemiological variables. We hypothesized that distinct genomic, population and phylogeographic signatures in SARS-CoV-2 circulate during different phases of the epidemic. We used a comprehensive collection of the SARS-CoV-2 genomes isolated from COVID-19 patients and accompanying epidemiological data identified within the GISAID database^13^. As a separate analysis, we coupled SARS-CoV-2 genomic sequences from GISAID with other complete coronavirus genomes from GenBank (“Methods”).

**Figure 1:**
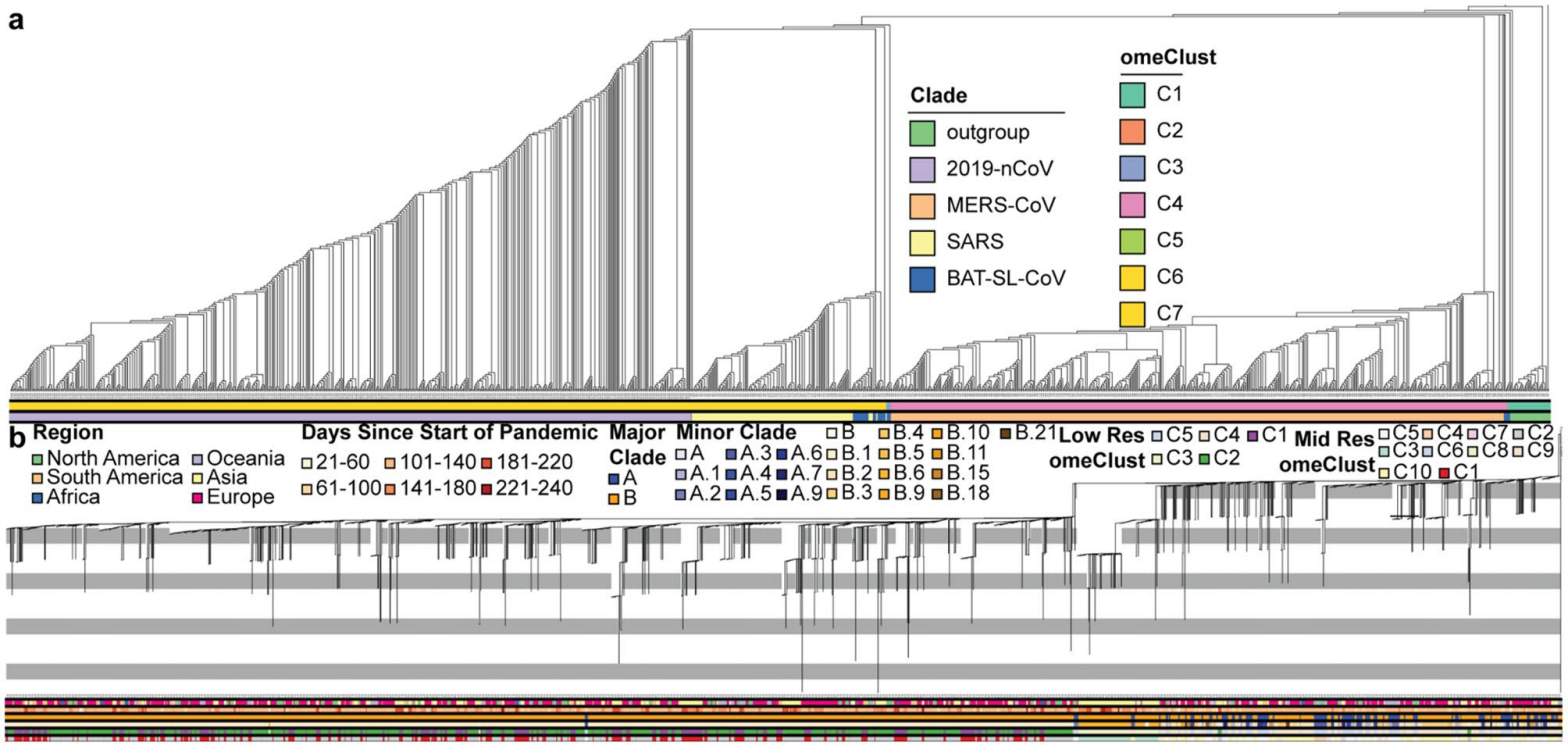
Maximum Likelihood analysis of the nsp3 region of the CoV genomes. (**a**) RAxML cladogram (branch lengths not proportional to change) showing relationships between SARS-CoV-2, MERS, Bat-SL-CoV, and SARS-related and rooted using a Beta Coronavirus outgroup. Sequence identity estimates between the representatives of clades for CoV families reveal regions with potential functional importance. (**b**) RAxML phylogram (branch lengths proportional to change) estimated from 2,007 sequences from the GISAID database, including proportional representatives of genomes from Pangolin major clades.

Our results indicate that the phylogenetic trees based on the sequence alignments of nsp3 (Fig. 1) and Spike protein (Supplementary Fig. 1) provide similar phylogenetic structure to the whole viral genome tree (Supplementary Fig. 2), although some clade differences were found for each coronavirus. We found that SARS-CoV-2, SARS-related, and MERS-CoV comprise distinct clades, with a SARS-related plus bat clade sister to SARS-CoV-2. Two groups of Bat-SL-CoV taxa were sister to MERS-CoV and interspersed with the SARS-related clade. The homology-based distance of the viral genome has been used to distinguish the clades using the *omeClust* approach.

The major clades depicted in our tree conform well with previously detected lineages^14^, but distinguished different minor lineages from those in Rambaut’s tree. Our phylogenetic analysis indicates that SARS-CoV-2 is sister to a clade comprised of bat coronavirus and SARS-related genomes. Bat-SL-CoV also falls in several clades sister to and within the SARS-related clade, and sister to the clade including SARS-related, SARS-CoV-2, and MERS-CoV. *omeClust* was able to identify four different lineages corresponding to four coronavirus families (normalized mutual information (NMI) = 0.9).

### CoV subclades, diversity, and gene signal

**Figure 2:**
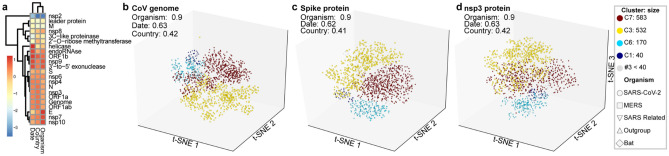
Subclade identification using CoV genome and gene variation in population of sample in our study. Subclade finding was performed using *omeClust* and enrichment score of metadata was measured based on the overlap of detected clades and metadata using normalized mutual information (NMI). (**a**) regions of CoV genome have been clustered using z-score of enrichment scores for three metadata variables available for all lineages. Regions such as S, nsp6, N, nsp3, ORF1a, ORF1ab are more similar to genomes using clusters of scaled enrichment scores. (**b**) *omeClust* identifies communities of CoV lineages that are mostly explained by organisms (NMI = 0.9). (**c**) Spike protein that facilitates binding and entering to host cells carries similar variation among organisms as the whole CoV genome. (**d**) nsp3 protein has a similar variation to S protein and can be targeted as a protein with an important biological function. *omeClust* detects four communities (points colors) corresponding to the four known organisms (points shapes).

To investigate the influence of the CoV genes in the phylogenetic trees, we applied *omeClust* to the inter-sample distances of 29 regions and CoV genomes from the GISAID SARS-CoV-2 alignment. We identified communities of CoV lineages by applying *omeClust* (“Methods”) to a distance matrix built from dissimilarity among genome alignments. Distance matrices were calculated for genome and gene partitions using GTR + G distances^15,16^. These distances are used as inputs into the *omeClust* algorithm along with clinical data including organism, date (year), and country. The correlation between CoV lineage communities and metadata reported by *omeClust* as enrichment score using normalized mutual information for all CoV genes is shown (Fig. 2a). Results show that variation in distance matrices for the genomes and specific regions is explained by metadata and the Spike protein and nsp3 regions correlate with the viral genome overall. Identified clades of CoV using genome variation (Fig. 2b) are explained mostly by organisms (NMI = 0.9). Date (NMI = 0.62) and country (NMI = 0.41) also have some influence in the clade structure. *omeClust* results identified clades and organisms as the most influential metadata and suggest that the major variation among coronavirus organisms happens in the Spike (Fig. 2c) and nsp3 proteins (Fig. 2d). *omeClust* gives similar enrichment scores for the whole genome, and the Spike protein and nsp3 region. However, regions such as E, nsp10, and M have different specifications and separation properties. In *omeClust* analysis, we used five distinguished organisms (“Methods”).

### Within-viral genome variation associations reveal important genes

**Figure 3:**
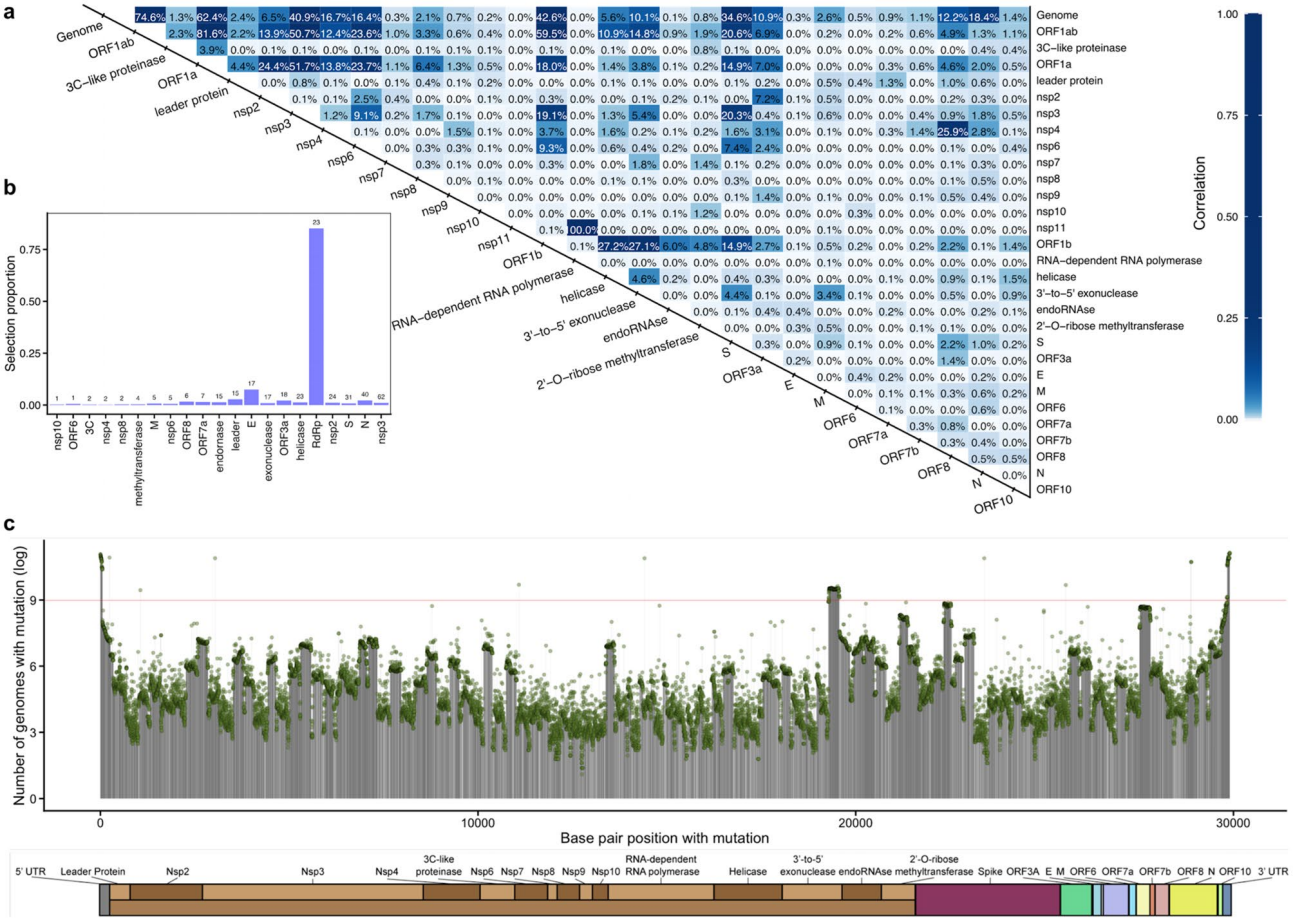
Association among SARS-CoV-2 genome and genes variation. (**a**) The SARS-CoV-2 genome and 29 specific regions are used to structure dissimilarity among samples in the GISAID cohort. Relationships between variation explained among proteins, regions, and the whole genome of CoV using paired measurements with differences across subjects are quantified by Mantel tests (square of the Mantel statistic, see “Methods”). (**b**) the selection proportion (histogram bars) and the number of sites under selection (values above the bars) for each of the 21 specific regions detected by HyPhy^17^ on October 28, 2020. Spike protein and nsp3 are among the regions with a high number of sites under selection, while nsp10 and ORF6 regions have the lowest number of sites. The RNA-dependent RNA polymerase (RdRP) has the highest selection proposition from the HyPhy analysis, the number of sites under selection divided by the length of the gene region, which shows no association in our analyses. (**c**) the count of SARS-CoV-2 SNPs (in log scale) shows distinct patterns across genome regions. The 3′-to-5′ exonuclease, endoRNAse, 2′-O-Ribose methyltransferase, and Spike proteins have a heightened number of mutations. The red line is an arbitrary cutoff at log(8000) to emphasize large differences as we show the results in the log scale.

To identify associations among CoV genes including well-studied genes (e.g., Spike protein) and other CoV genes, we used the correlation between nucleotide level distances in population samples. We generated distance matrices for our study samples from 2069 representative GISAID samples using homology dissimilarity (TN93 model distances) across 29 specific genome regions (“Methods”). Then to investigate the relationship between region variation, we tested if there was a relationship between the overall structure of these dissimilarity matrices using the Mantel test^15^. This is an important step to relate proteins with clinical outcomes and identify mechanisms in COVID-19 genotype–phenotype associations such as Spike protein (S) variant G614 having an evolutionary fitness advantage compared to D614^16^ that we also see in our data in location the 23,403 A > G. We used 29 specific regions of the viral genome and the whole genome. Our results indicate that variation in the whole genome is significantly associated with variation in the Spike protein (correlation = 0.32) and nsp3 regions (correlation = 0.39) (Fig. 3a). Additionally, the Spike protein is associated with nsp3 (correlation = 0.20), which is the highest correlation between the Spike protein and individual gene regions/proteins (excluding metaregions ORF1ab and the full genome), and nsp6 (correlation = 0.07), which has a potential role for facilitating viral replication^10^. Genome regions that are not associated with any other regions need to be investigated individually with metadata of interest.

We used the number of selected sites as a characterization of change in each site of the viral genome to prioritize potential important regions. Consistent with our population-level variation, the Spike protein and nsp3 have high numbers of sites under selection (Fig. 3b). We obtain the number of sites under selection from HyPhy^17^, which is embedded in Datamonkey 2.0^18^. In addition, we investigated mutations across the CoV2 genomes (“Methods”). Sites flagged as mutations at the beginning and end of the genome are likely sequencing artifacts and not veritable mutations (Fig. 3c). The 3′-to-5′ exonuclease, endoRNAse, 2′-O-Ribose methyltransferase, and Spike proteins—roughly from bp 19,000 to 23,000—show a greater-than-average frequency of mutations. The two peaks of mutation counts in nsp3 (location 3037 C > T) and Spike protein (location 23,403 A > G) tend to co-occur^19^.

### Associations among SARS-CoV-2 genes and epidemiological data

**Figure 4:**
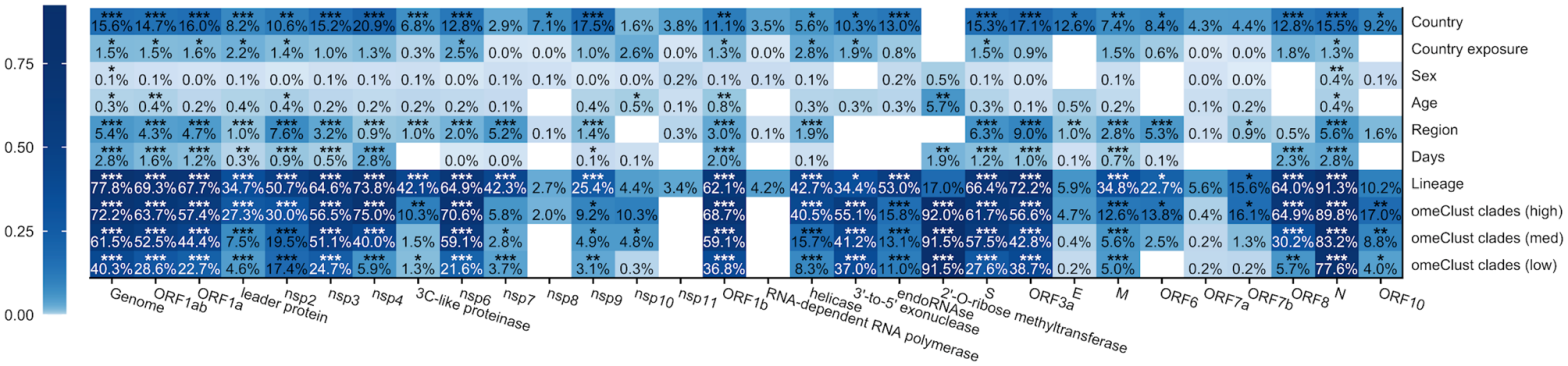
Association between SARS-CoV-2 genome regions and metadata. Distances among CoV genomes and 29 specific regions using GTR + G-based distances were used to assess relationships between variation explained between proteins, regions, and the whole genome of SARS-CoV-2 using paired measurements with differences across subjects by omnibus (PERMANOVA) test. White cells refer to scenarios where there was not sufficient variation to perform our analyses.

We explored associations between viral genome similarity across samples and metadata downloaded from GISAID (“Methods”) using the PERMANOVA test (Fig. 4). Our results indicated host characteristics such as Age and Sex are less correlated with the viral genome variation compared to other epidemiological data such as Country and Days. Association between days, country, country of exposure, and region with SARS-CoV-2 genome and genes indicates different CoV clades of the SARS-CoV-2 are spreading in different world regions and the virus is evolving over time.

We used lineage information provided in the GISAID as a control for our analysis as it is driven by viral genome variation and we expected a significant correlation between lineage and genome variation. Viral communities detected by *omeClust* and lineage reveal similar associations with CoV genome and genes suggesting alignment between two approaches, lineage labeling, and *omeClust* community detection.

### SARS-CoV-2 variation and geography

**Figure 5:**
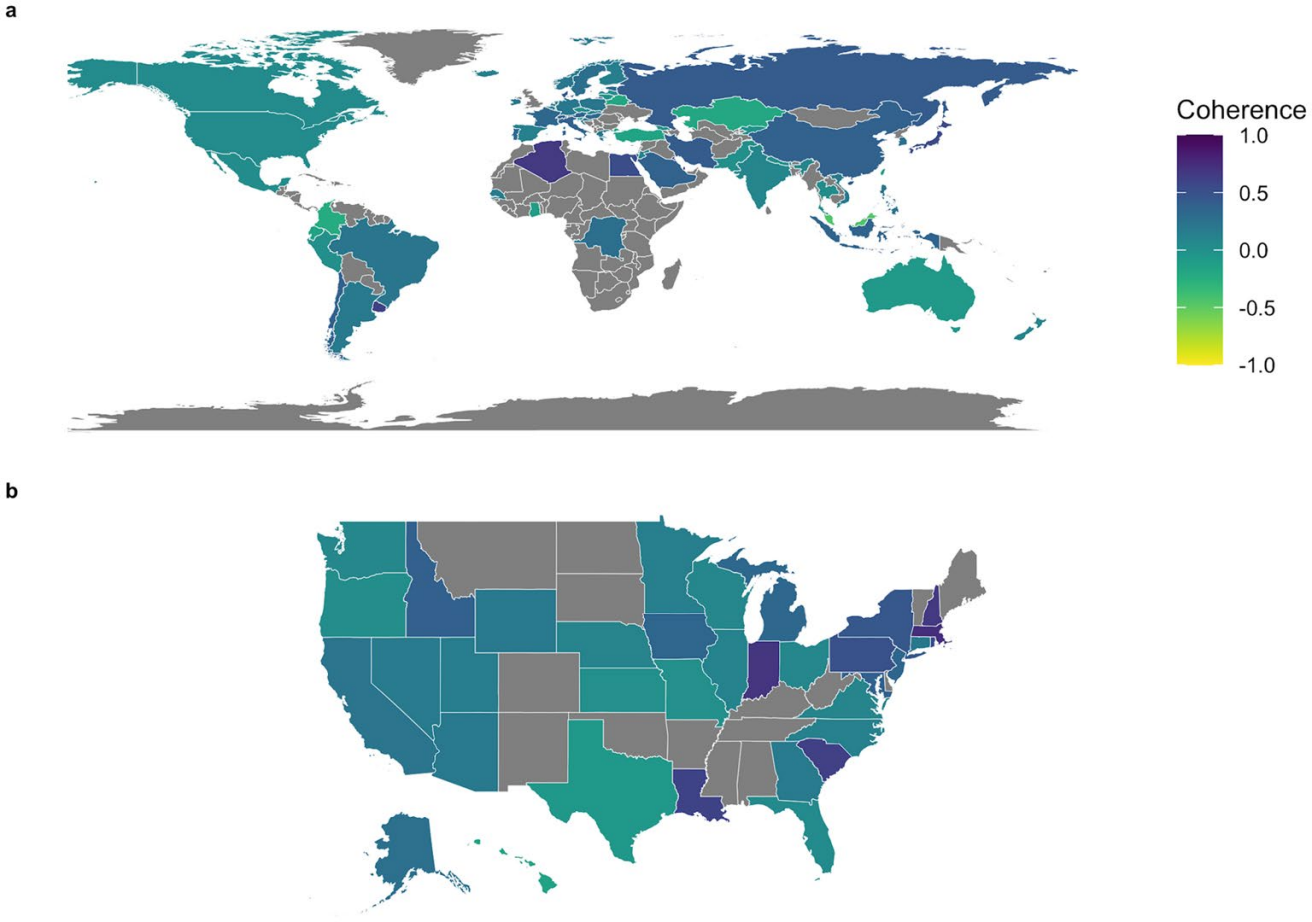
Quantification of coherence of lineages within a specified area compared to other areas. Higher coherence values indicate lower phylogenetic distance within a specific geographic region relative to other areas. (**a**) 15,721 viral genome sequences from infected individuals downloaded from GISAID on May 8th, 2020, and the sequencing data were aligned and used to compare the diversity of SARS-CoV-2 within countries compared to the rest of the world. (**b**) samples from each state of the US have been compared to the rest of the US to investigate the similarity of the virus lineages within each state. Several counties and states exhibited differentiation into specific clades. States or countries with darker colors likely show a higher level of community-driven spread. In contrast, states or countries with lower coherence (lighter colors) show a greater level of disease introduction from outside the region. The figure is implemented in *omicsArt*^22^, a ggplot2^23^ based R^24^ package.

We used the viral genome alignment among our samples to investigate the diversity and spatiotemporal distributions of SARS-CoV-2 lineages. Location-wide phylogenetic distances indicate the homogeneity of lineages in specified locations (countries world-wide, and states for the USA). We defined a ‘coherence’ measurement to quantify the similarity of lineages within a location (countries or states) compared to other locations (“Methods”). Countries with greater coherence show greater similarity and less phylogenetic separation of lineages (Fig. 5). The coherence can be used as a measurement of diversity and spatiotemporal distributions of SARS-CoV-2 lineages in a region of interest. A coherence score of 1.0, like in Qatar, indicates that viral lineages in Qatar are more similar to each other compared to the rest of the world. Brunei, Algeria, Kuwait, Uruguay, Egypt, and Japan follow behind Qatar, with coherence scores greater than 0.5. A high within-community diversity is indicated by a low coherence score such as Malaysia (coherence = − 0.45). A natural extension for this metric is in the realm of public health, where coherence can be used as a surrogate for assessing the effectiveness of policies such as the use of face coverings^20^ and/or travel restrictions^21^ (Fig. 5b).

### Genomic variation related to protein structures

Protein structures have important roles in viral function (e.g., binding to human cells) and provide an additional dimension for understanding viruses, especially relative to their evolution to drug and vaccine resistance. We used the SWISS model^25^ to build protein structures of coronavirus families using available RefSeq genomes for Bat-SL-CoV, SARS, MERS-CoV, and SARS-CoV-2. Our results suggest that there are clear differences between important proteins across coronavirus families. For example, the Spike protein has a different structure in SARS-related vs. Bat-SL-CoV and MERS-CoV; however, it has a similar structure to SARS-CoV-2 (Supplementary Fig. 3). The Spike protein has important functions such as facilitating entry into target cells^26^ via host attachment and virus-cell membrane fusion, determining host range^27^, and lipid modification^10^. Spike protein variation was significantly correlated with nsp3 (coefficient = 0.20); here, we show a similar pattern for nsp3 in predicted protein structure, where SARS-related and SARS-CoV-2 have similar nsp3 secondary structures, as well as Bat-SL-CoV and MERS-CoV, which also have similar nsp3 secondary structures.

We used proteins that have a high correlation with genome variation, Spike protein, and nsp3 protein, and four proteins with lower variation, including N, ORF6, 2′ O Ribose Methyltransferase proteins. Our results suggest that ORF6 is the most variable region in terms of secondary structure. The S protein is dominated by coils in its secondary structure.

**Figure 6:**
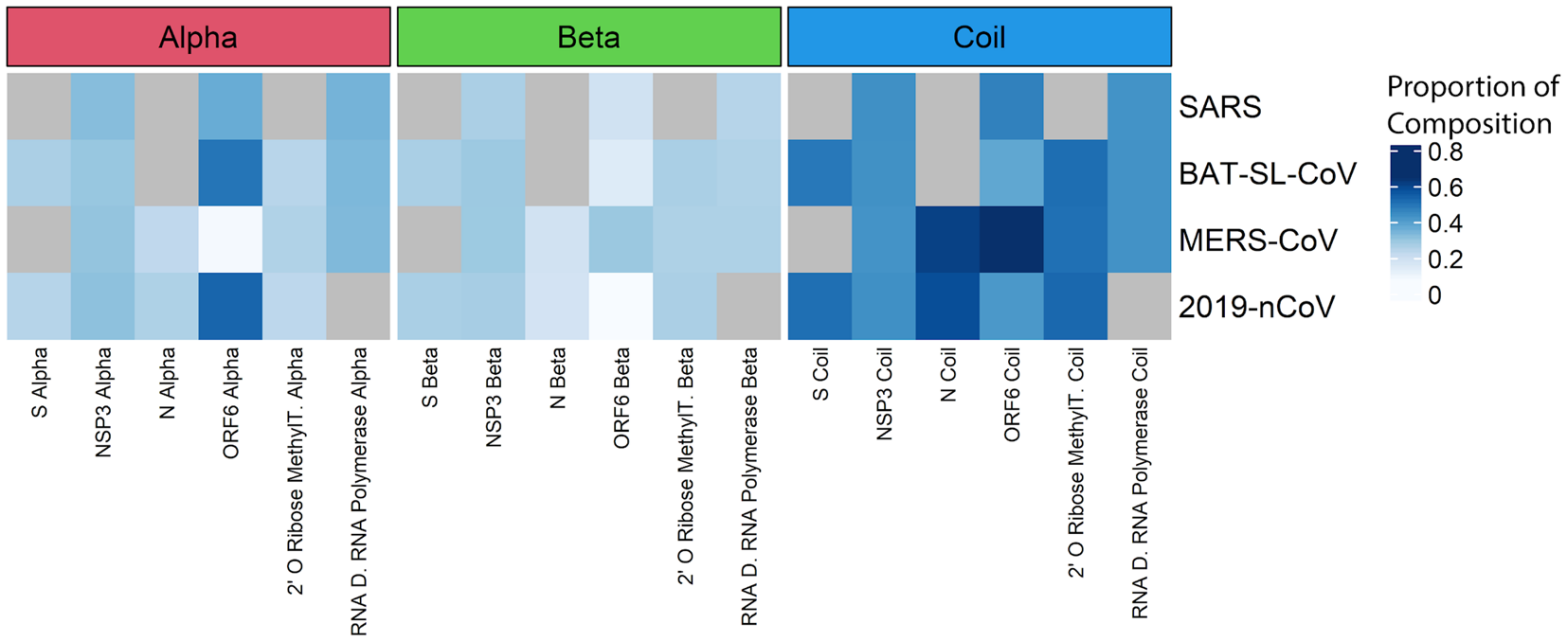
Predicted protein structure from sequence data across coronavirus families. (**a**) proteins with high variation among coronaviruses tend to have different protein structures. Blank cells indicate proteins that could not be successfully modeled by SWISS-MODEL (**b**) amino acid composition in predicted secondary structures of proteins show different patterns among CoV genome proteins. Gray cells refer to proteins that contained stop codons in our alignment or were otherwise not amenable to structural analysis.

Protein secondary structures come in three classes: helices, strands, and coils. Different secondary structures show different robustness to mutations; in this context, robustness refers to the ability of a molecule to maintain its shape or function when its residues are mutated. A feature with higher robustness can have a greater number of mutations without undergoing structural change. Proteins composed of helices have been shown to be more robust to mutations than strands and both are more robust than coils (Fig. 6). In other words, proteins that are composed of helices are more likely to retain their folded conformation when their residues are mutated^28^. It is, therefore, interesting that coils, the least robust of the three structural features, dominate the structure of most proteins in the betacoronavirus family. Mutations, by this logic, are more likely to have a high structural impact in these viruses. A notable exception is ORF6 alpha of SARS-CoV-2, which shows a high proportion of helices compared to other proteins across the different viruses.

## Discussion

SARS-CoV-2 virus has infected individuals across the world, and no demographics and individual characteristics have shown immunity to the infection. The outcome of COVID-19 disease has varied among individuals and, as the virus evolves rapidly, characterizing this variation sheds light on understanding viral behaviors. We investigated viral genomic variation relative to genomic features (e.g., sites under natural selection, gene regions, etc.), epidemiological features (e.g., date of diagnosis, age, sex, region, etc.), and protein structural features (e.g., coils, helices, loops, etc.)—although our approach can be used for other clinical features (e.g., obesity, coinfections, drug treatments, etc.) when they become available. We used two paths of viral genome investigations comparing: (1) variation among coronavirus types such as MERS-CoV and Bat-SL-CoV, and SARS-CoV-2 to find differences amongst these related lineages of coronaviruses and associated phenotypes (e.g., speed of spread and severity of the disease), and (2) variation among SARS-CoV-2 genomes to variants associated with differences in clinical and epidemiological data.

We hypothesized that specific genes that give a higher enrichment score (separation) of viral lineages might have an important role in viral behavior, such as transmissibility and severity, and this can be further explained by testing their associations with clinical metadata. We applied variation association tests not only to the whole genome alignment data, but also to 29 specific regions of the viral genome, to obtain sharper resolution of the potential function of important variation. We consistently found that Spike protein and nonstructural protein 3 (nsp3) significantly represent the viral genome variation among coronavirus families and also among SARS-CoV-2 genomes. We additionally highlight sites undergoing selection in the N gene; the origin of this selective pressure may come from the role of the nucleocapsid protein in producing nuclear localization signals^29^. We also identify mutational hotspots in the 3′-to-5′ exonuclease, endoRNAase, 2′-O-Ribose methyltransferase, Spike protein, ORF7a/b, and ORF8. Mutations in some regions have been linked to host functional alterations such as the role of ORF7a in host immune suppression^30^ and ORF8’s role in intracellular stress pathways^31,32^. Functional roles of other viral mutations remain to be characterized. We assessed associations between distances among genes with epidemiological features and found genetic variation correlated with country of exposure and days from start of the pandemic. Our community finding approach using dissimilarity from viral genomes and regions found subclades consistent with GISAID lineage data. Our coherence analysis framework provides an insightful lens to examine viral evolution in a geographic and public health context. We observed striking differences across states, even states which are contiguous to one another. For example, Idaho has a remarkably different coherence score compared to Oregon and Washington, while Louisiana’s score is drastically different from that of Texas. This trend is similarly observed at a more global level—China and Kazakhstan, for example, have dramatically different coherence scores. Travel, face covering usage, local restrictions, and other factors may play a role in these differences and additional investigation is warranted to characterize viral spread and containment via genomic features. Regardless, the results suggest that coherence may serve as an important population level measurement for designing and assessing public health efforts.

In the future, we need to pair viral genome variation to host metabolomics and protein changes to provide a more detailed description of biological activity as host responses. This requires paired omics, metabolite and protein profiling, and also the characterization of the human microbiome, as well as well-designed studies to investigate specific aspects of virus infections and related health outcomes. For example, confounding factors (e.g., ethnicity and previous health records) need to be considered. Our quantifications of important genes (proteins) enable generating hypotheses for COVID-19 treatment, such as vaccine development and gene editing in targeted regions of the viral genome. Overall, we present a compelling analytical framework for integrating clinical data and viral genomic data to make novel discoveries and insights on the interactions between these data types, all of which provides helpful insights into combating COVID-19.

## Methods and materials

### Viral genome sequences

We downloaded SARS-CoV-2 genomic sequences from GISAID and aligned them with other complete coronavirus genomes from GenBank for bat coronavirus (Bat-SL-CoV, including Bat coronavirus, Bat SARS coronavirus HKU3, and Bat SARS-like coronavirus), Middle East Respiratory SARS-related coronavirus (MERS-CoV), Severe acute respiratory syndrome-related coronavirus (SARS-related), and outgroup sequences from the *Alphacoronavirus* genus^33^ from the transmissible gastroenteritis virus. We segmented both the SARS-CoV-2 genome alignment and the combined coronavirus genome alignment into 29 segments corresponding to the gene regions of SARS-CoV-2. In our analyses, we used a set of coronavirus genomes as a representative of phylogenetic tree including genomes for all previously recorded coronavirus genomes (e.g., MERS-CoV, and Bat-SL-CoV) in GenBank and a representative set of SARS-CoV-2. We kept the number of SARS-CoV-2 here as large as MERS-CoV genomes, the largest set we have for other coronavirus families. We downloaded a representative set of SARS-CoV-2 genomes with complete metadata (including age and sex, year, and country) from individuals infected during the COVID-19 pandemic from the GISAID database. These data were used for testing associations between variation in SARS-CoV-2 genomes and available epidemiological metadata.

### Sequence alignment and phylogeny estimation

For our dataset containing only SARS-CoV-2 sequences, no alignment was necessary as the MSA was available from GISAID. This alignment was subsetted to 2069 representative genomes of all clades for ease of computational analysis. Subsetting was performed so that each clade would be represented proportionally in the smaller dataset, this was accomplished computationally by calculating the proportional size of each clade, multiplying that number by 500, rounding up, and then randomly sampling the resulting number of sequences from each clade:

$$ Number\;To\;Select = \left\lceil {500*\frac{number\;of\;sequences\;in\;clade}{{total\;number\;of\;sequences}} } \right\rceil $$

For the combined data set with SARS-CoV-2 (580 genomes) + Bat-SL-CoV + MERS-CoV + SARS-related + outgroup sequences (a total of 1,339 sequences), full-length genome sequences were aligned using the global alignment strategy in MAFFT^34^ via the CIPRES Science Gateway V. 3.3^35^ under the default parameters.

From these alignments, we used Modeltest^36^ to infer the best model of evolution for alignments from both datasets, and these sequences were used to reconstruct a phylogeny in RAxML using a rapid bootstrap analysis and the GTRGAMMAX model^37^. RAxML-HPC BlackBox on the CIPRES Science Gateway V. 3.3^35^ was used with the *mlsearch* and *bootstrapping* parameters. After estimating phylogenies, several long branches were observed and subsequently trimmed to account for significant variance in sequence quality. Branches with a length of > 0.0006 were trimmed and removed from the alignment, as were sequences with a proportion of > 0.035 of ‘N’ characters in the genome. We performed these filtering steps under the assumption that those branches were likely assigned long lengths or an erroneous placement in the tree due to low-quality sequencing runs. We segmented aligned genomes into 29 individual coding and non-coding regions based on the features from the GenBank entry NC_045512.2, the SARS-CoV-2 reference genome. The GenBank entry contains the basepair position start and end points of each of these 29 regions; that information was used to divide the alignment into constituent regions. Upon dividing, phylogenies were reconstructed for two genes of particular interest—nsp3 and S. Phylogenies were estimated using maximum likelihood as implemented in RAxML as described above. We extracted the SNPs from the multialignment file of the CoV genomes using the snp-sites tool^38^. Then we kept sites with a minor allele frequency greater than 0.00005 using vcftools (v0.1.13)^39^ with maf threshold = 0.00005.

To investigate associations among variation in viral genome regions and with epidemiological data, we performed analyses on both whole-genome viral alignments as well as regional alignments. The multiple sequence alignment (MSA) was subsequently divided into 29 constituent gene regions (i.e., spike, envelope, ORF1A, etc.) by fetching the GenBank entry for the SARS-CoV-2 reference sequence, splitting the genomes based on the base pair positions that correspond to constituent gene regions, and outputting a single multi-fasta file for each gene region. In so doing, we generated 29 multi-fasta files, each containing the sequence for an individual gene region for each of the sequences in our MSA file. If our MSA contained 500 sequences, then the script would generate 29 multi-fasta files, each containing 500 sequences. Each of these files was then subjected to a distance calculation using the *dna.dist* function in the R^24^ package ape^40^. Our code is available at https://github.com/omicsEye/covid-19/.

### *OmeClust* for community detection

Our *omeClust* algorithm (http://github.com/omicsEye/omeClust) proceeds by (1) building a representation of the overall structure of the viral samples based on their genome similarity (a hierarchy) and hierarchical clustering (zoom out), (2) descending the hierarchy to find heterogeneous clusters (zoom in) using a binary-silhouette score, (3) calculating resolution score for each cluster to prioritize important clusters and enrichment score for each metadata to rank the impact of the metadata on the detected communities. Dissimilarity matrices between the CoV genome regions were calculated using the TN93 model from the R package ape^40^ between samples using both whole viral genomes and specific protein-coding genes of the CoV-2 genome. Then, we applied *omeClust* on each dissimilarity matrix along with metadata and clinical information collected for all samples. *omeClust* produces two main outcomes: 1) it detects the communities or groups of samples (community structure) and 2) determines the influential metadata variables in association with the discovered structural groups (subclades). For each variable, it calculates an enrichment score using the normalized mutual information score between sample community labels and sample metadata.

### Mantel test

The Mantel test statistic (the Pearson correlation between distances) is used to quantify correlation among distance matrices between samples in populations. *mantel.test* function in *ape* R package was used for this analysis.

### PERMANOVA test

PERMANOVA^41^ was used to measure variance explained by metadata of interests (e.g., age, gender, and country) using the adonis function in the R package *vegan*. The total variance explained by added metadata including lineage and three resolutions (low, mid, and high) of *omeClust* variables were calculated independently. Then, the total variance explained of other variables, including country, country of exposure, sex, age, regions, and days were calculated dependently, including all other variables in the model. We used the omnibus (PERMANOVA) test to detect associations between SARS-CoV-2 genome variation (CoV lineages, genes, and genomes) and epidemiological (e.g., date of diagnosis, age, sex, race, region, etc.) data.

### Coherence measurement

Similarity of SARS-CoV-2 lineages within locations (e.g., countries and states) compared to other locations were detected by a ‘coherence’ measurement defined here. Coherence is a measure based on the silhouette score^42^, in a supervised manner where sample labels are used as cluster labels for which we then calculate the silhouette score for each label. In this study, lineages from one location are considered as members of one cluster. The coherence approach compares the mean phylogenetic divergence of lineages within each location to the divergence of lineages (within the same location) traversing in all locations. For each location, the silhouette score is used to quantify how members of a cluster are heterogeneous within one location compared to the rest of the locations in the study. The resulting coherence score, then, is on a scale from -1 to 1 with 0 indicating no coherence (i.e., relatively equal phylogenetic diversity within locations compared to among locations), 1 would indicate phylogenetic diversity at a given location is extremely low relative to surrounding phylogenetic diversity (perhaps indicative of a very isolated founding event), whereas -1 suggests more phylogenetic diversity within the location compared to among locations, which would be indicative of diversification within location.

### Mutations across CoV2 genomes

We extracted the SNPs from the multialignment file of the CoV genomes using the snp-sites tool^38^. Then we kept sites with a minor allele frequency greater than 0.00005 using vcftools (v0.1.13)^39^ with maf threshold 0.00005. For this analysis, we aligned 15,721 CoV2 genomes (downloaded from GISAID on May 8th, 2020) to a reference CoV2 genome from Wuhan using the MAFFT tool.

### Protein structure analysis and SWISS model

Using the gene partitioning schema previously described, we identified the amino acid sequence using each of the three possible reading frames for each gene region across MERS-CoV, SARS-related, Bat-SL-CoV, and SARS-CoV-2 genomes. Six proteins—S, nsp3, N, ORF6, 2′ O Ribose Methyltransferase and the RNA Dependent RNA Polymerase were chosen for structural analysis. The first two were identified as variable regions, the other 4 were more consistent. The sequences without stop codons were inferred to be viable proteins and modeled using the interactive modeling module on SWISS-MODEL. In the case where multiple models were successfully generated for a given amino acid sequence, the model with the highest Global Model Quality Estimation (GMQE) score was selected. Images were captured with the “Take Snapshot of 3d Molecule” option using “extreme” as the resolution selection.

Amino acid sequences were subsequently analyzed using the predict HEC function in the R package DECIPHER^43^, which calculates the probability of a residue forming part of a helix, beta-sheet, or coil conformation using the GOR IV method^44^. A window size of 7 was used. The predicted secondary structure was then further analyzed to calculate the proportion of each of the aforementioned conformations in each residue. The results of this analysis were presented as heatmaps using the *pheatmap* package in R.

## Supplementary Information

Supplementary Information.
