# Supplementary material for: Epidemiological associations with genomic variation in SARS-CoV-2
Source: Sci Rep. 2021 Nov 26;11:23023. doi: 10.1038/s41598-021-02548-w (PMC8626494; doi:10.1038/s41598-021-02548-w)
Supplement: Supplementary file 1 — Supplementary Information. [file 41598_2021_2548_MOESM1_ESM.pdf]

Supplementary Figures for

Epidemiological associations with genomic variation in SARS-CoV-2

Ali Rahnavard[1]*, Tyson Dawson[1], Rebecca Clement[1], Nathaniel Stearrett[1], Marcos Pérez-Losada[1,2], Keith A. Crandall[1]

[1] Computational Biology Institute, Department of Biostatistics and Bioinformatics, Milken Institute School of Public Health, The George Washington University

[2] CIBIO-InBIO, Centro de Investigação em Biodiversidade e Recursos Genéticos, Universidade do Porto, Campus Agrário de Vairão, Vairão, Portugal

*Correspondence to rahnavard@gwu.edu

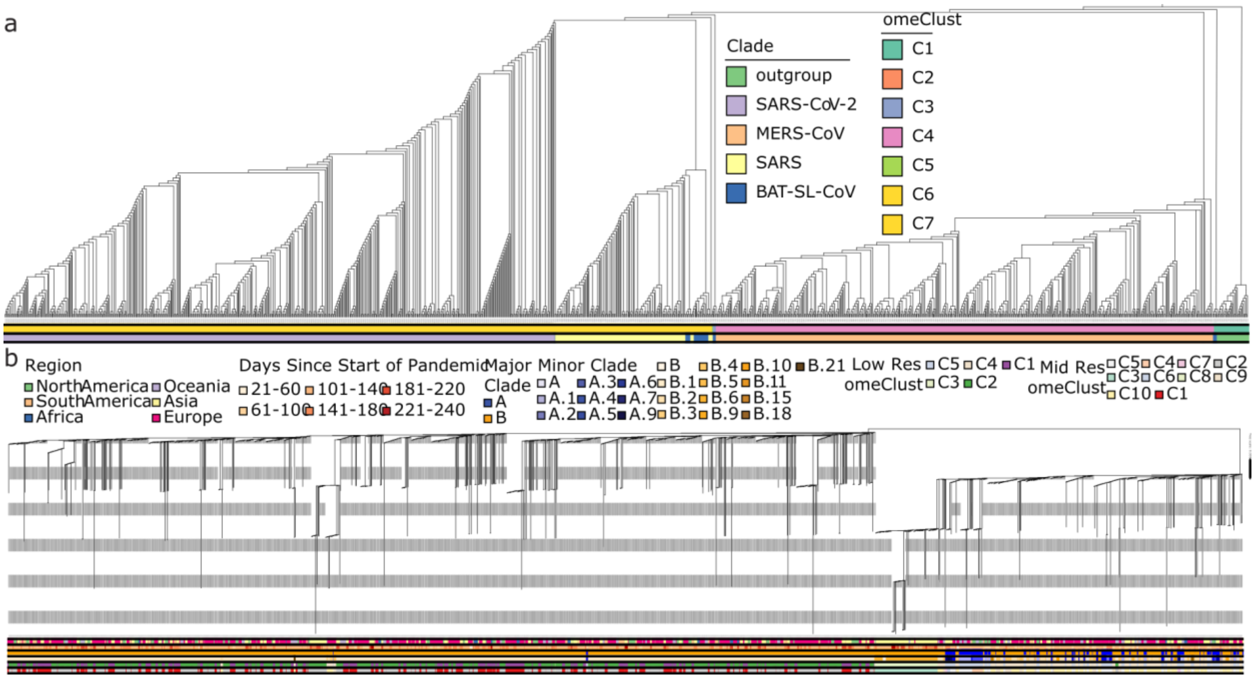

Supplementary Figure 1: Maximum Likelihood analysis of Spike gene of CoV genomes. a, RAxML tree showing relationships between SARS-CoV-2, MERS, Bat-SL-CoV, and SARS-related rooted to a Beta Coronavirus outgroup. Branch lengths are not shown. Sequence identity between the representatives of strain clades for CoV families reveals regions with potential functional importance. **b**, RAxML tree built from 2,007 sequences from the GISAID database with proportional representatives of genomes from Pangolin major clades with Spike gene.

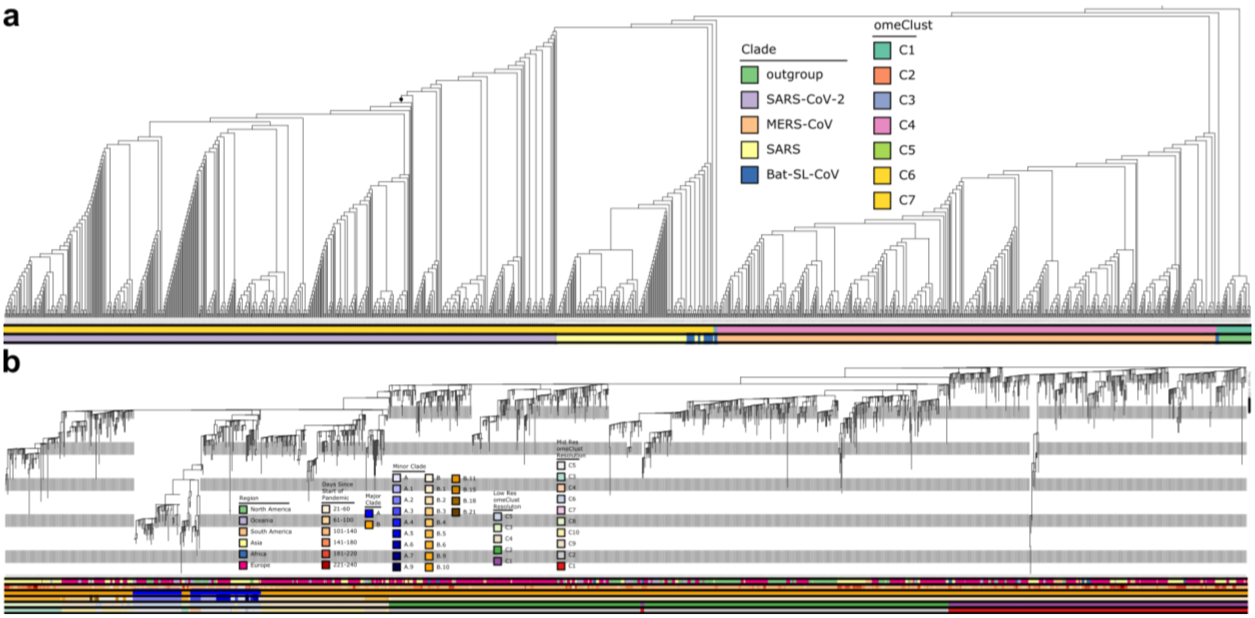

Supplementary Figure 2: Maximum Likelihood analysis of the whole CoV genomes. a, RAxML tree showing relationships between SARS-CoV-2, MERS, Bat-SL-CoV, and SARS-related rooted to a Beta Coronavirus outgroup. Branch lengths are not shown. Sequence identity between the representatives of strain clades for CoV families reveals regions with potential functional importance. **b**, RAxML tree built from 2,007 sequences from the GISAID database with proportional representatives of genomes from Pangolin major clades with the whole viral genome.

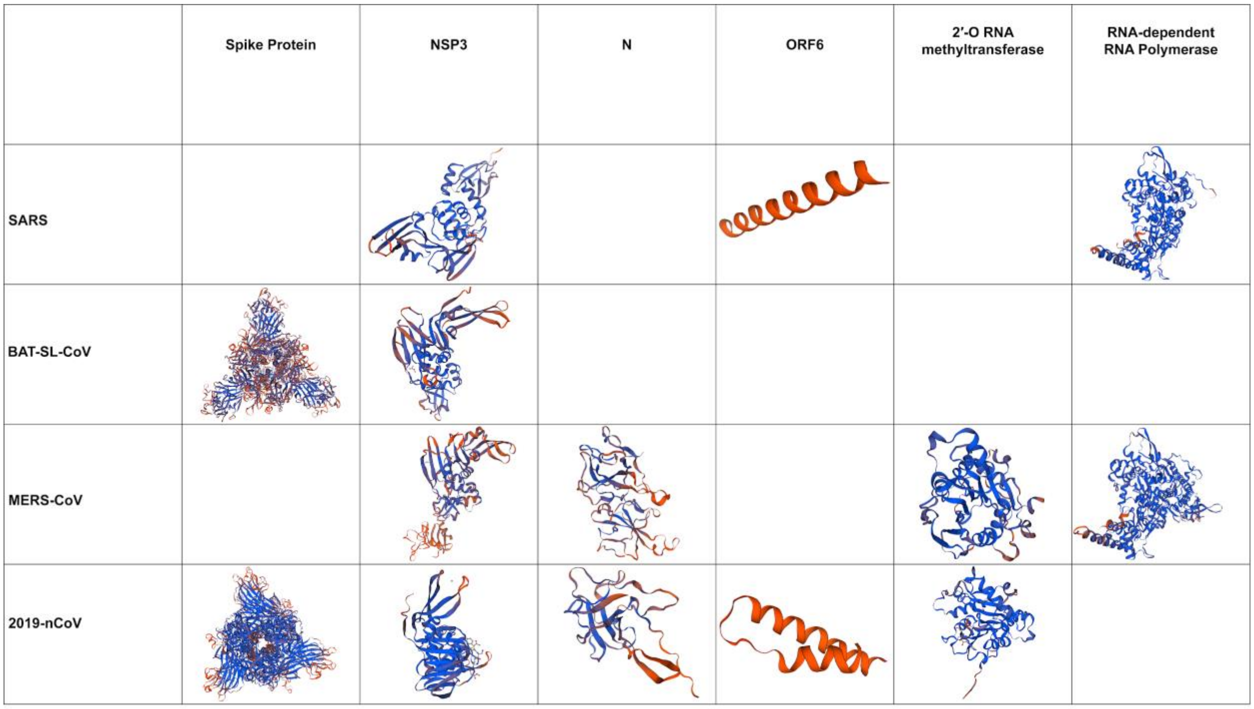

Supplementary Figure 3: Predicted protein structure from sequencing data across coronavirus families. Proteins with high variation among coronaviruses tend to have different protein structures. Blank cells indicate proteins that could not be successfully modeled by SWISS-MODEL.
